# Surgical Strategy of Intravenous Leiomyomatosis with Intracardiac Extension: A Case Report

**DOI:** 10.3400/avd.cr.25-00062

**Published:** 2025-08-28

**Authors:** Shun Sato, Kazuo Yamanaka, Yuri Hashimura, Michiyuki Ichikawa, Yuichi Tara, Daisuke Nakatsuka, Takeshi Nishina

**Affiliations:** Department of Cardiovascular Surgery, Nara Prefecture General Medical Center, Nara, Nara, Japan

**Keywords:** intravenous leiomyomatosis (IVL), intracardiac extension, cardiac tumor

## Abstract

Intravenous leiomyomatosis with intracardiac extension is a rare benign tumor originating from uterine smooth muscle. A 50-year-old woman presented with a cardiac mass 3 years after hysterectomy. Imaging revealed a tumor extending from the right internal iliac vein to the right atrium. Complete resection was achieved via a 2-stage surgery. In the 1st stage, median sternotomy and a retroperitoneal approach were performed, and the intracardiac tumor was excised under deep hypothermic circulatory arrest with cardiopulmonary bypass. Postoperatively, gonadotropin-releasing hormone (GnRH) agonist therapy was administered, followed by a 2nd-stage resection of the residual pelvic tumor and right ovary. The patient remained recurrence-free for 15 months.

## Introduction

Intravenous leiomyomatosis (IVL) is a rare disease in which a leiomyoma arising from uterine smooth muscle or uterine vein smooth muscle develops intravascularly. Histologically, IVL is a benign tumor, but it may extend into the inferior vena cava (IVC) or cardiac cavity, resulting in fatal complications such as tricuspid valve impaction or pulmonary embolization. We experienced a case of resection of IVL that extended from the right internal iliac vein through the IVC into the right heart chamber. As we completely resected the IVL with a 2-stage operation, we presented our surgical strategy, focusing on the 1st-stage operation.

## Case Report

**Fig. 1:**
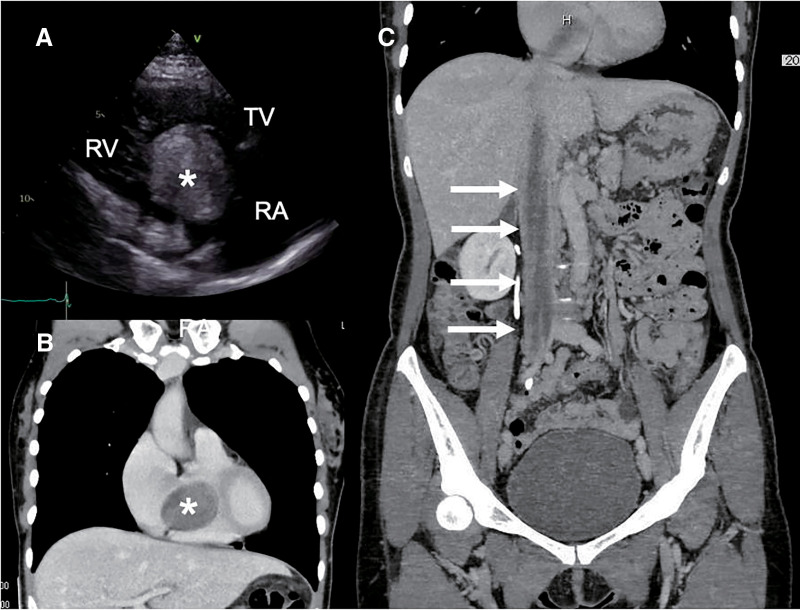
Transthoracic echocardiography and computed tomography. (**A**) Transthoracic echocardiography revealed the presence of a spherical mass (asterisk), measuring 30 × 30 mm, within the right heart chambers, which was moving across the TV. (**B**, **C**) Computed tomography demonstrated a mass (asterisk) extending from the right internal iliac vein to the right heart chamber via inferior vena cava (arrows). RA: right atrium; RV: right ventricle; TV: tricuspid valve

A 50-year-old woman visited the gynecology department with a 3-month history of dyspnea and fatigue. Initially suspected to be experiencing menopausal symptoms, she was prescribed medication, which failed to alleviate her condition. Transthoracic echocardiography (TTE) was performed, and a cardiac tumor was suspected; she was later referred to our hospital. At the age of 47, she had undergone a simple hysterectomy and right adnexectomy for uterine myoma. Physical examination revealed no leg edema or heart murmur. TEE showed a spherical mass, 39 × 45 mm in size (**Fig. 1A**), in the right heart chamber that moved back and forth through the tricuspid orifice into the right ventricle. The mass was linear and extended into the IVC. Contrast-enhanced computed tomography (CT) demonstrated a mass extending from the right internal iliac vein to the right heart chamber via IVC (**Figs. 1B** and **1C**). On pelvic contrast-enhanced magnetic resonance imaging (MRI), a residual mass was present near the right ovary, from which the mass was continuous to the internal iliac vein and extended into the common iliac vein. She was diagnosed with IVL with intracardiac extension. As there was a risk of tricuspid valve inversion, urgent resection was indicated.

**Fig. 2:**
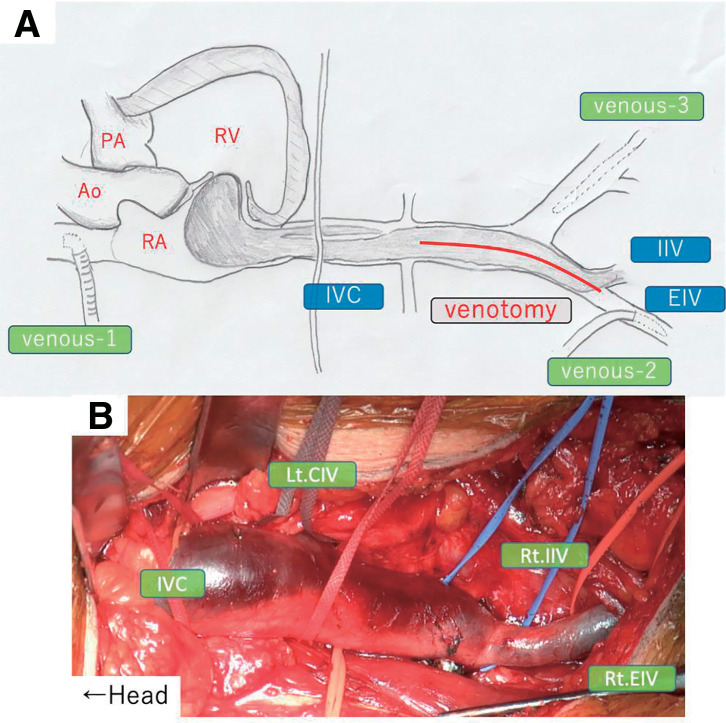
Surgical procedure. (**A**, **B**) The surgery was performed with cardiopulmonary bypass by cannulating the ascending aorta, with venous drainage through the superior vena cava, right external iliac vein, and left femoral vein. The tumor was divided at the renal vein level, and tumor resection was completed through right atriotomy and venotomy under deep hypothermic circulatory arrest at 20°C. Ao: aorta; EIV: external iliac vein; IIV: internal iliac vein; IVC: inferior vena cava; Lt: left; PA: pulmonary artery; RA: right atrium; Rt: right

Surgery was performed through a median sternotomy (medial approach) and a right-sided oblique incision (right retroperitoneal approach) because of severe pelvic adhesions based on prior surgical findings. We installed cardiopulmonary bypass via a cannula in the ascending aorta and venous cannulas in the superior vena cava, right external iliac vein, and left femoral vein, and started cooling until a deep hypothermia at 20°C (**Fig. 2**). First, the right retroperitoneal approach provided access to the IVC. The bilateral common iliac veins and right internal and external iliac veins were exposed and taped, and a hard mass was palpated in the IVC. Next, the IVC with intravenous tumor and left common iliac vein were clamped, and the IVC was incised in the long axis direction under core cooling. The tumor in the IVC was divided at the renal vein level; the peripheral side was resected down to the left internal iliac vein. The internal iliac vein was ligated and dissected. Its stump was sealed with a bovine pericardial patch. Other iliac veins were reconstructed with a bovine patch.

Then, the right atrium was incised into the IVC under circulatory arrest, and the remaining tumor was excised. The tumor was easily resected manually despite mild adhesions. A biliary endoscope confirmed the absence of residual tumor at the hepatic vein level. After resuming circulation, rewarming was initiated. The tricuspid valve annulus was dilated by the tumor, and tricuspid annuloplasty was performed using a 26-mm Tri-Ad Adams tricuspid ring (Medtronic, Minneapolis, MN, USA). The total cardiopulmonary bypass time was 184 min, aortic cross-clamp time was 37 min, and circulatory arrest time was 11 min. She was extubated on the 1st postoperative day and discharged from the hospital on the 12th postoperative day.

**Fig. 3:**
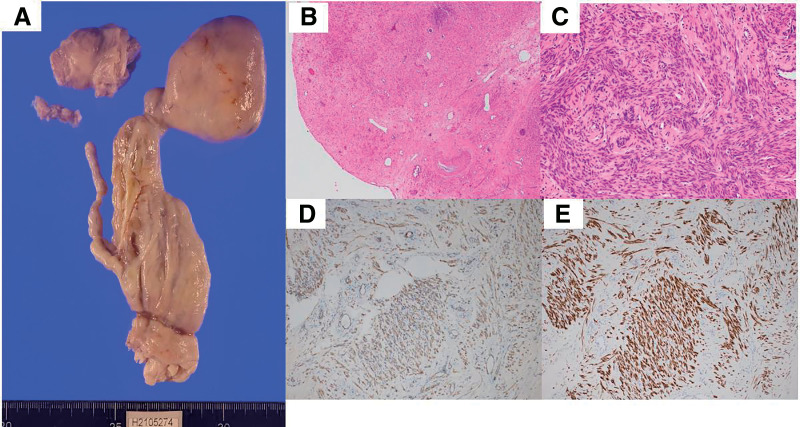
Specimen. The operative specimen consists of an intracardiac spherical component and a 30-cm-long intravenous component (**A**). Histopathological findings. H&E staining (**B**: ×40; **C**: ×200) shows bundles of spindle-shaped smooth muscle cells. Immunohistochemical staining for α-SMA (**D**) and desmin (**E**) demonstrates cytoplasmic positivity. α-SMA: α-smooth muscle actin; H&E: hematoxylin and eosin

The resected specimen consisted of a spherical tumor, measuring 50 × 70 mm, connected to a 30-cm-long tumor (**Fig. 3A**). The tumor was pale pink, smooth, elastic, and firm. Histological examination revealed spindle-shaped tumor cells without mitosis or necrosis, with strong immunohistochemical staining for α-smooth muscle actin and desmin (**Figs. 3B**–**3E**).

On the 22nd postoperative day, the patient was started on gonadotropin-releasing hormone (GnRH) agonist-based hormonal therapy by the gynecologist. Nine months after surgery, resection of the tumor around residual uterine myoma tissue and the right ovary was performed. She had no recurrence for 15 months.

## Discussion

IVL is a rare benign neoplastic disease with intravenous extension of uterine myoma or leiomyoma arising from the uterine vein. IVL is a female-specific disease with a predilection for the 40- to 50-year-old age range.^1)^ It is characterized by the presence of previous surgery for uterine myoma or untreated uterine myoma in more than 80% of patients. In the present case, the patient had undergone a total hysterectomy and right adnexectomy for uterine myoma 3 years prior. The residual tumor was presumed to have developed in the ovarian vein, subsequently extending into the IVC and right heart.

The clinical manifestations of IVL are variable and nonspecific. When tumors extend into the right heart, they can lead to potentially fatal outcomes, such as syncope or sudden cardiac death.^2,3)^

The standard treatment for IVL with intracardiac extension is surgical resection under cardiopulmonary bypass with deep hypothermia and circulatory arrest through median sternotomy and midline laparotomy. It has been reported that about one-third of patients with incompletely resected tumors develop recurrence,^4)^ so complete resection of the tumor is important.^5)^ Although some reports suggest that resection of small, localized tumors may be accomplished through sternotomy or laparotomy alone, there is also a case indicating that traction on the tumor through sternotomy alone led to fatal retroperitoneal hemorrhage. Therefore, the operative approach requires careful consideration.^6)^

The choice between 1- and 2-stage resection remains controversial. One-stage surgery is typically preferable when the patient is in good general condition and the tumor’s progression route is uncomplicated. However, based on a joint meeting of gynecologists and cardiovascular surgeons, we chose a 2-stage resection and right retroperitoneal approach because of the presence of severe intraperitoneal adhesions observed during the previous surgery and the necessity for extensive dissection. To prevent recurrence of tumor extension into the IVC, the right internal iliac vein was completely ligated, and its stump was sealed with a bovine pericardial patch.

In this case, although the tumor was benign and did not infiltrate the IVC wall, preoperative imaging suggested the possibility of tumor fragmentation or thrombus attachment. Therefore, we used a biliary endoscope intraoperatively to confirm complete tumor removal. Although the IVC was collapsed during circulatory arrest and visibility was limited, the scope could be advanced to the IVC incision site, and no residual tumor was identified.

Even if the tumor had been malignant, we believe it could have been resected using the same technique. In general, given the risk of recurrence, a 1-stage resection is preferable whenever feasible. For tumors with suspected invasion of the IVC wall at the hepatic vein level, the Pringle maneuver or liver mobilization may be required to achieve complete resection. However, such procedures involve significant surgical invasiveness, and their indications should be carefully considered.

Hormone therapies, such as anti-estrogen and GnRH agonist therapy, have been reported to shrink the tumor in some cases of IVL^7)^ and may be useful in preventing recurrence or managing incomplete resections.

## Conclusion

We report a case of IVL extending into the right heart, in which the tumor was resected via a 2-stage surgery. It is crucial to select an appropriate surgical approach that takes into account the patient’s overall health, tumor location, and the extent of its vascular involvement.
